# Weight loss improves disease activity in patients with psoriatic arthritis and obesity: an interventional study

**DOI:** 10.1186/s13075-019-1810-5

**Published:** 2019-01-11

**Authors:** Eva Klingberg, Annelie Bilberg, Sofia Björkman, Martin Hedberg, Lennart Jacobsson, Helena Forsblad-d’Elia, Hans Carlsten, Björn Eliasson, Ingrid Larsson

**Affiliations:** 1 Department of Rheumatology and Inflammation Research, Sahlgrenska Academy at the University of Gothenburg, Gothenburg, Sweden; 2 Institute of Neuroscience and Physiology, Section of Health and Rehabilitation, Physiotherapy, Sahlgrenska Academy at the University of Gothenburg, Gothenburg, Sweden; 3 Department of Gastroenterology and Hepatology, Sahlgrenska University Hospital, Gothenburg, Institute of Medicine, Sahlgrenska Academy at University of Gothenburg, Gothenburg, Sweden; 4 Department of Rheumatology, Hospital of Borås, Borås, Sweden; 5 Department of Public Health and Clinical Medicine, Rheumatology, Umeå University, Umeå, Sweden; 6 Department of Medicine, Institute of Medicine, Sahlgrenska University Hospital, University of Gothenburg, Gothenburg, Sweden

**Keywords:** Psoriatic arthritis, Psoriasis, Obesity, Metabolic syndrome, Weight loss, VLED, Cardiovascular disease

## Abstract

**Background:**

Obesity is over-represented in patients with psoriatic arthritis (PsA) and associated with higher disease activity, poorer effect of treatment and increased cardiovascular morbidity. Studies on the effects of weight loss are however needed. This study aimed to prospectively study the effects of weight loss treatment with very low energy diet (VLED) on disease activity in patients with PsA (CASPAR criteria) and obesity (body mass index BMI ≥ 33 kg/m^2^).

**Methods:**

VLED (640 kcal/day) was taken during 12–16 weeks, depending on pre-treatment BMI. Afterwards, an energy-restricted diet was gradually reintroduced. Weight loss treatment was given within a structured framework for support and medical follow-up.

Treatment with conventional synthetic and/or biologic disease-modifying anti-rheumatic drugs was held constant from 3 months before, until 6 months after baseline.

Patients were assessed with BMI, 66/68 joints count, Leeds enthesitis index, psoriasis body surface area (BSA), questionnaires and CRP at baseline, 3 and 6 months. Primary outcome was the percentage of patients reaching minimal disease activity (MDA) and secondary outcomes were reaching Psoriatic Arthritis Response Criteria (PsARC) and American College of Rheumatology (ACR) response criteria.

**Results:**

Totally 41/46 patients completed the study, 63% women, median age 54 years (IQR 48–62). At baseline increased BMI was associated with higher disease activity and poorer function.

The median weight loss was 18.7 kg (IQR 14.6–26.5) or 18.6% (IQR 14.7–26.3) of the baseline weight. A majority of the disease activity parameters improved significantly after weight loss, including 68/66 tender/swollen joints count, CRP, BSA, Leeds enthesitis index, HAQ and patient VAS for global health, pain and fatigue. A larger weight loss resulted in more improvement in a dose-response manner. The percentage of patients with MDA increased from 29 to 54%, (*p* = 0.002). PsARC was reached by 46.3%. The ACR 20, 50 and 70 responses were 51.2%, 34.1% and 7.3% respectively.

**Conclusions:**

Short-term weight loss treatment with VLED was associated with significant positive effects on disease activity in joints, entheses and skin in patients with PsA and obesity. The study supports the hypothesis of obesity as a promotor of disease activity in PsA.

**Trial registration:**

ClinicalTrials.gov identifier: NCT02917434, registered on September 21, 2016—retrospectively registered

**Electronic supplementary material:**

The online version of this article (10.1186/s13075-019-1810-5) contains supplementary material, which is available to authorized users.

## Background

Psoriasis affects 2–3% of the population in Sweden and 20–30% of individuals with psoriasis develop psoriatic arthritis (PsA), a chronic rheumatic disease leading to synovitis, enthesitis and sometimes also axial involvement [1]. Both psoriasis and PsA are strongly associated with obesity and the metabolic syndrome (MetS) [2]. International observational studies have reported prevalence of obesity (body mass index (BMI) ≥ 30 kg/m^2^) in 45% of PsA patients and of MetS in 30–45% [3–5]. Previous studies have shown that obesity increases the risk of developing both psoriasis and PsA and that obesity is associated with higher disease activity, poorer treatment response and lower chance of achieving minimal disease activity (MDA) [3, 6–10]. Patients with PsA also have an increased risk of cardiovascular disease (CVD) [11–13]. Chronic inflammation, which accelerates the atherosclerotic process, in combination with higher prevalence of cardiovascular (CV) risk factors among PsA patients is believed to contribute to this increased risk [14–16].

Although the associations between PsA and obesity, BMI and disease activity are well known, there is to the best of our knowledge only one prior interventional study which has prospectively assessed the effects of weight loss treatment in PsA [17].

The aim of this study was to determine the effects of weight loss treatment with very low energy diet (VLED) on the disease activity in joints, entheses and skin in patients with PsA and obesity. The primary endpoint of the study was the percentage of patients reaching minimal disease activity (MDA) after 6 months weight loss treatment. Secondary endpoints were the percentage of patients reaching American College of Rheumatology (ACR) and Psoriatic Arthritis Response Criteria (PsARC).

## Patients and methods

### Patients

Patients with PsA and obesity registered at the Rheumatology clinics of Sahlgrenska University hospital and the hospitals of Alingsås and Borås were invited to participate.

Patients with PsA fulfilling the Classification for Psoriatic Arthritis (CASPAR) criteria, with a BMI ≥ 33 kg/m^2^ and age 25–75 years were eligible for inclusion [18]. If using conventional synthetic and/or biologic disease-modifying anti-rheumatic drugs (cs and/or bDMARDs), the treatment had to be constant and unchanged from 3 months prior to baseline until 6 months after baseline. Exclusion criteria were pregnancy, porphyria, epilepsy, type 1 diabetes, severe heart, kidney or catabolic disease, binge eating disorders, treatment with warfarin, lithionin or phenantoin, mental imbalance affecting participation, being subject to a heart infarction, stroke, major surgery or trauma during last the 3 months and being treated for cancer during the last 5 years.

All the patients in the study gave their written informed consent. The study was approved by the Regional Ethics Committee in Gothenburg and carried out in accordance with the Helsinki Declaration.

### Intervention: weight loss treatment with very low energy diet (VLED)

Weight loss treatment with VLED (< 800 kcal/day) is an effective and established method in clinical use in Sweden. In severe obesity, BMI ≥ 35.0 kg/m^2^, a rigorous energy restriction is needed for optimal weight loss [19]. The VLED used in the present study provided a daily intake of 640 kcal including recommended doses of vitamins, minerals and other essential nutrients (Cambridge Weight Plan Limited, Corby, UK). The diet consisted of four daily portions of powder dissolved in cold or hot water and consumed as shakes or soups. Based on pre-treatment BMI (< 40 or ≥ 40 kg/m^2^), the participants followed an initial period of 12 or 16 weeks on strict VLED. In addition, non-energy-containing beverages were allowed at libitum*.* During VLED, the mean weight loss usually is 1–2 kg per week. Adherence to treatment was controlled by measuring the body weight of the patients regularly. Absent or limited weight loss was considered a sign of poor adherence to the diet.

After the strict period, food was gradually reintroduced during a period of 12 weeks. Each patient was instructed to follow an energy-restricted diet based on individual energy requirements for weight stability reduced by 30% to achieve further weight loss. The side effects of the VLED treatment are mostly mild to moderate and transient and ceases when the strict period is ended. The most prevalent side effects are headache, nausea, obstipation, dryness of skin and hair loss [19]. The treatment is not associated with any health risks, when given under the surveillance of health professionals following an evidence-based protocol [19, 20]. The treatment was given within the framework of a 12-month protocol including structured weight loss treatment, support and medical follow-up from a team of doctors, nurses and dieticians at the Obesity Unit at Sahlgrenska University Hospital.

### Measures of assessment

All examinations were done at baseline, after 3 months and 6 months. No blinding of assessors was made. Body height and weight were measured with a calibrated ruler and digital scale. Waist circumference was measured with a tape measure in centimetres midway between the lower rib and iliac crest. Joints were examined with 66/68 swollen/tender joints count and entheses with Leeds enthesitis index [21]. The extent of psoriasis was evaluated with body surface area (BSA) [22]. Quality of life related to psoriasis was assessed with the Dermatology Life Quality Index (DLQI) [23]. The patients’ disease activity was assessed with visual analogue scales (VAS) for global disease activity, pain and fatigue. The physician’s global assessment of disease activity was also evaluated with a VAS. Function and activity limitations were assessed using the Health Assessment Questionnaire (HAQ) [24]. Both the Disease Activity Score using 28 joint counts based on CRP (DAS28CRP) and the Disease Activity in PSoriatic Arthritis (DAPSA) score were calculated [25, 26].

The primary endpoint minimal disease activity (MDA) is defined as meeting five of the seven following criteria: tender joint count 68 ≤ 1, swollen joint count 66 ≤ 1, psoriasis body surface area ≤ 3%, patient pain VAS ≤ 15 mm, patient global disease activity VAS ≤ 20 mm, HAQ ≤ 0.5 and tender entheseal points ≤ 1 [27].

Secondary endpoints were the number of patients reaching the PsA Response Criteria (PsARC) and the American College of Rheumatology (ACR) 20, 50 and 70 criteria for treatment response. The PsARC is defined as improvement in at least two of the following four areas: ≥ 20% improvement in physician’s global VAS, ≥ 20% improvement in patient global disease activity VAS, ≥ 30% improvement in 66 swollen joint count, ≥ 30% improvement in 68 tender joint count. Improvement in the swollen or tender joint counts is mandatory, and there should be no worsening of any component [28, 29]. The ACR 20, 50 and 70 criteria are defined as a 20%, 50% or 70% improvement in the 28 swollen or tender joint counts together with a 20%, 50% or 70% improvement in at least 3/5 of the following: the patient’s global health VAS, patient’s pain VAS, physician’s global VAS, HAQ and CRP or ESR [30].

Blood samples were analysed for haemoglobin (Hb), white blood cell count (WBC), platelet count (PLT) and C-reactive protein (CRP) using standard laboratory techniques at Sahlgrenska University Hospital.

### Statistical analyses

Statistical analyses were made using SPSS Statistics version 25 (IBM, Chicago, USA). Descriptive statistics are presented as median and interquartile range (IQR) and range. Wilcoxon signed rank test was used to compare continuous related samples and McNemar test to compare categorical related samples. Correlations were calculated using Spearman’s correlation (*r*_S_). All tests were two-tailed, and *p* ≤ 0.05 was considered statistically significant.

## Results

### Characteristics of the study population

In total, 46 patients were included and started VLED treatment, whereof five patients (11%) dropped out before the 6 months visit (at the beginning of the VLED treatment, one cancelled participation and one was excluded due to depression, after 3 months two cancelled participation and one was excluded due to pregnancy).

A total of 41 patients completed the study, 63% women. The median baseline age was 54 (IQR 48–62) years. The characteristics of the study population are shown in Table 1.

**Table 1:** Characteristics of the 41 patients with PsA and obesity included in the study

	Number (%) or median (IQR)
Sex women/men, *n* (%)	26 (63.4)/15 (36.6)
Age, years	54 (48.5–62)
Duration of psoriasis, years	32 (19–40)
Duration of PsA symptoms, years	17 (11–27)
PsA type, *n* (%)	
Peripheral disease	35 (85)
Axial disease	2 (5)
Combination peripheral/axial	4 (10)
History of dactylitis, *n* (%)	21 (51)
History of anterior uveitis, *n* (%)	3 (7)
NSAIDs, *n* (%)	27 (66)
TNFi all, *n* (%)	15 (37)
TNFi in monotherapy	4
TNFi + csDMARD	11
Ustekinumab monotherapy, *n* (%)	1 (2.4)
csDMARD without biologic, *n* (%)	19 (46)
Methotrexate	13
Sulfasalazine	2
Apremilast	1
Methotrexate + sulfasalazine	3
Anti-hypertensives, *n* (%)	18 (44)
Lipid lowering therapy, *n* (%)	6 (15)
Oral anti-diabetics, *n* (%)	1 (2.4)

*csDMARD* conventional synthetic disease modifying anti-rheumatic drug, *NSAID* non-steroidal anti-inflammatory drug, *TNFi* tumour necrosis factor inhibitor

### The association between BMI and disease activity at baseline

At baseline BMI was positively correlated with several measures of disease activity and function including DAS28CRP, DAPSA, tender joints count, CRP, patient’s global health VAS, Leeds enthesitis index and HAQ (Spearman’s rho ranged from *r*_S_ = 0.312, *p* = 0.047 to *r*_S_ = 0.483, *p* = 0.001) (Table 2).

**Table 2:** The correlation between BMI and disease activity and function at baseline in patients with PsA and obesity (*N* = 41)

	Spearman’s rho	*p* value
CRP, mg/L	0.312	0.047
Tender joints 68, score	0.333	0.034
Swollen joints 66, score	− 0.139	0.385
VAS patients global disease activity, mm	0.370	0.017
VAS pain, mm	0.298	0.059
VAS fatigue, mm	0.220	0.168
DAS28CRP, score	0.382	0.014
DAPSA, score	0.360	0.021
Leeds enthesitis index, score	0.483	0.001
BSA, %	−0.155	0.334
HAQ, score	0.457	0.003
DLQI, score	−0.113	0.483

*BMI* body mass index, *BSA* body surface area, *CRP* C-reactive protein, *DAPSA* Disease Activity in PSoriatic Arthritis, *DAS28CRP* Disease Activity Score using 28 joint counts based on CRP, *DLQI* Dermatology Life Quality Index, *VAS* visual analogue scale

### Weight loss and waist circumference

All the patients lost weight, from a minimum of 8.5 to a maximum of 40.2 kg. The median weight loss from baseline to the 6 months visit was 18.7 kg (IQR 14.6–26.5), corresponding to a loss of in median 18.6% (IQR 14.7–26.3) or min–max range 8–35% of baseline weight. BMI decreased from median 35.2 kg/m^2^ (IQR 34.1–38.1) to 29.7 kg/m^2^ (IQR 26.2–31.5) (*p* < 0.001) and waistline circumference was reduced from median 116 cm (IQR 112–122) to 95.5 cm (89–103) (*p* < 0.001).

### Change in disease activity

**Fig. 1:**
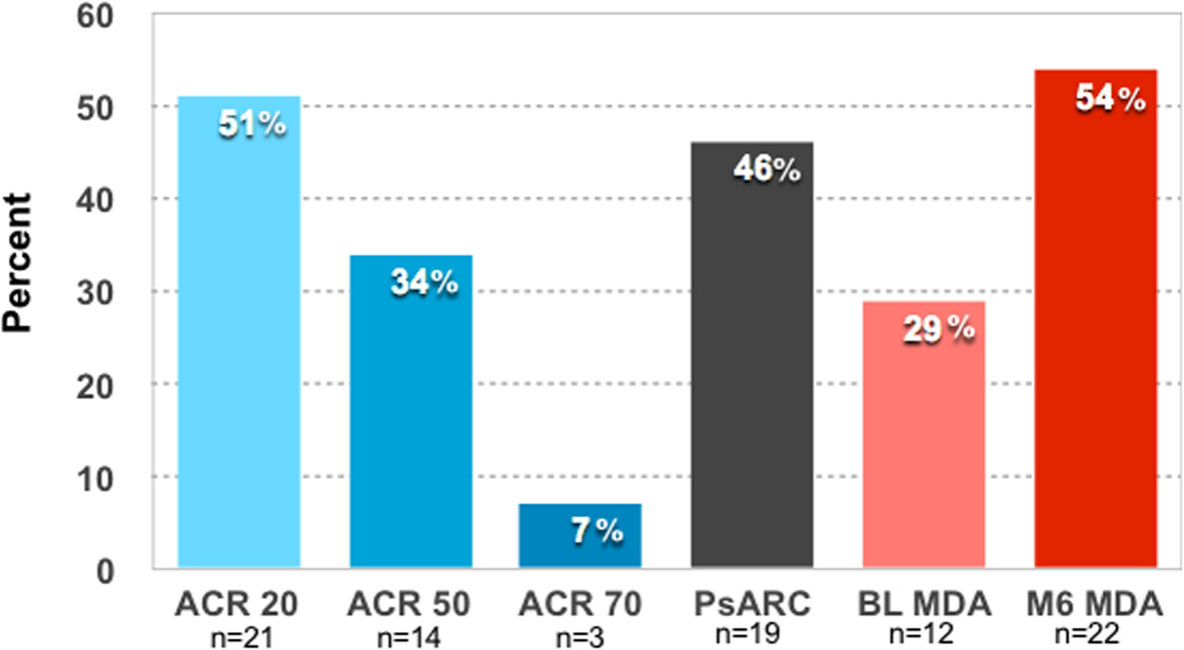
The percentage of patients reaching American College of Rheumatology (ACR) 20, ACR 50 and ACR 70 response criteria, Psoriatic Arthritis Response Criteria (PsARC) and Minimal Disease Activity (MDA). BL, baseline; M6, 6 months visit

A significant reduction was seen in a majority of the disease activity measures at the 6 months visit (Table 3). The percentage of patients with MDA (primary endpoint) increased from 29.3% (*n* = 12) at baseline to 53.7% (*n* = 22) at 6 months (*p* = 0.002). Regarding the secondary endpoints, PSARC was reached by 46.3% (*n* = 19) of the patients and ACR 20, 50 and 70 responses by 51.2% (*n* = 21), 34.1% (*n* = 14) and 7.3% (*n* = 3) respectively (Fig. 1).

**Table 3:** Disease activity and function before and after weight loss treatment in 41 patients with PsA

	Baseline Median (IQR) Min–max	6 months Median (IQR) Min–max	*p* value
Weight, kg	106.3 (95.8–113.6) 84–124	82.9 (76.4–92.1) 61.3–107.4	< 0.001
BMI, kg/m^2^	35.2 (34.1–38.1) 33.0–45.8	29.8 (26.6–31.5) 23.1–37.0	< 0.001
Waist circumference, cm	116 (112–122) 103–135	95.5 (89–103) 81–116	< 0.001
CRP, mg/L	4 (2–8.5) 1–50	2 (1–6.5) 1–50	0.041
Hemoglobin, g/L	144 (132–150) 116–179	141 (131.5–148) 118–161	0.047
WBC, 10^9^/L	5.9 (5.2–7.6) 4.3–15.2	5.9 (5.0–6.8) (3.2–12.7)	0.062
PLT, 10^9^/L	270 (204–300) 174–444	230 (186–290) 156–402	< 0.001
Tender joints 68, score	4 (1–14) 0–30	2 (0–6.5) 0–19	< 0.001
Swollen joints 66, score	0 (0–1) 0–5	0 (0–0.5) 0–7	0.021
VAS patients global disease activity, mm	34 (19–61) 0–93	12 (5–51) 0–95	0.001
VAS pain, mm	30 (18.5–62.5) 0–95	20 (5–51.5) 0–95	0.004
VAS fatigue, mm	56 (21.5–67) 0–94	25 (8–44) 0–98	0.001
DAS28CRP, score	2.9 (2.1–3.7) 1.4–5.6	2.4 (1.7–3.0) 1.2–4.5	< 0.001
DAPSA, score	15.3 (6.6–29.1) 0.9–46	11.0 (2.8–17.6) 0.2–35	< 0.001
Leeds enthesitis index	1 (0–4) 0–6	0 (0–3) 0–4	0.001
BSA, %	1.6 (0–2.2) 0–10	0.9 (0–1.1) 0–5.5	0.014
HAQ, score	0.70 (0.13–1.00) 0–2.63	0.43 (0–0.69) 0–1.88	< 0.001
DLQI, score	1 (0–4.5) 0–17	1 (0–4) 0–20	0.453

*BMI* body mass index, *BSA* body surface area, *CRP* C-reactive protein, *DAPSA* Disease Activity in PSoriatic Arthritis, *DAS28CRP* Disease Activity Score using 28 joint counts based on CRP, *DLQI* Dermatology Life Quality Index, *PLT* platelet count, *WBC* white blood cell count

**Fig. 2:**
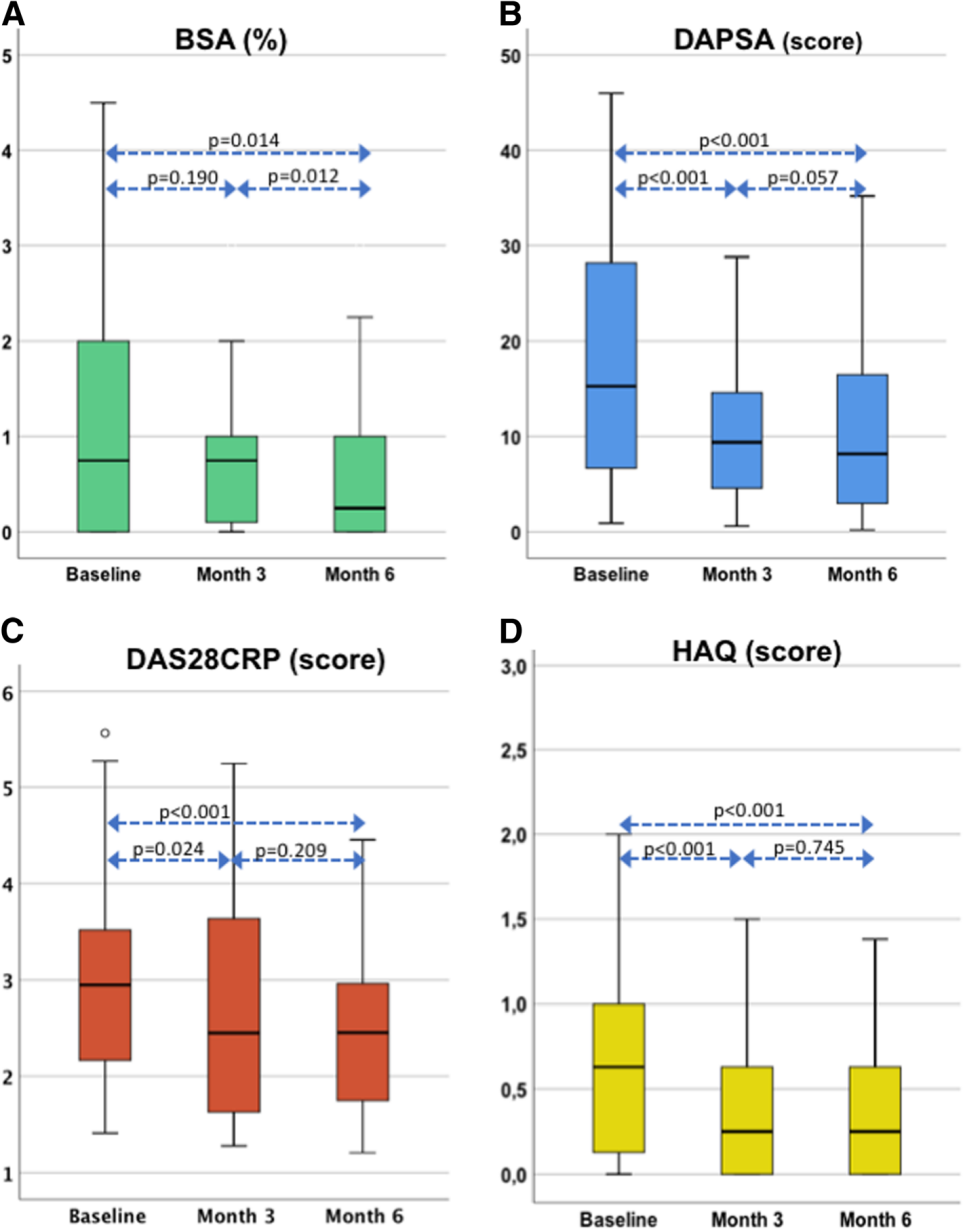
Boxplots showing the distributions at baseline and the 3 and 6 months visits of **a** psoriatic body surface area (BSA), **b** Disease Activity in PSoriatic Arthritis (DAPSA), **c** Disease Activity Score using 28 joint counts based on CRP (DAS28CRP) and **d** Health Assessment Questionnaire (HAQ)

The distributions of BSA, DAS28CRP, DAPSA and HAQ at baseline and the three- and six-months visits are shown in Fig. 2. The improvement of the skin occurred later than for the other disease activity parameters. Five patients (12%) experienced a flare in their psoriatic lesions at the 3 months visit. Six patients (15%) had a worsening of joint disease defined as an increase in DAPSA score (range + 1.9–9.1) at the 6 months visit. The laboratory parameters haemoglobin, CRP and PLT all declined significantly, compared with baseline.

A sub-analysis on the 29 patients who did not already have MDA at baseline, showed that PsARC was reached by 51.7% (*n* = 14) and ACR 20, 50 and 70 response by 62.1% (*n* = 18), 37.9% (*n* = 11) and 6.9% (*n* = 2) respectively of patients in this group. Disease activity and function before and after weight loss treatment in patients who did not have MDA at baseline is displayed in Additional file 1: Table S1.

### Improvement of disease activity in relation to grade of weight loss

The weight loss in percentage of baseline weight was positively correlated with Δ-DAS28CRP (*r*_S_ = 0.464; *p* = 0.002) and Δ-HAQ (*r*_S_ = 0.376; *p* = 0.015), but not with other Δ-values of disease activity. In addition, the ACR 20 response rate was significantly higher in patients with a weight loss ≥ median 18.6% (*n* = 21) compared with < 18.6% (*n* = 20) (ACR 20 71% vs 30%; *p* = 0.008). Similarly, a trend towards higher ACR 50 response rate was observed in patients with a weight loss ≥ 18.6% compared with < 18.6% (ACR 50 48% vs 20%; *p* = 0.062). The percentage reduction in waist circumference was also significantly correlated with the reduction in DAS28CRP (Δ-value) (*r*_S_ = 0.374; *p* = 0.019).

### Side effects and the patients’ experiences of the VLED treatment

**Fig. 3:**
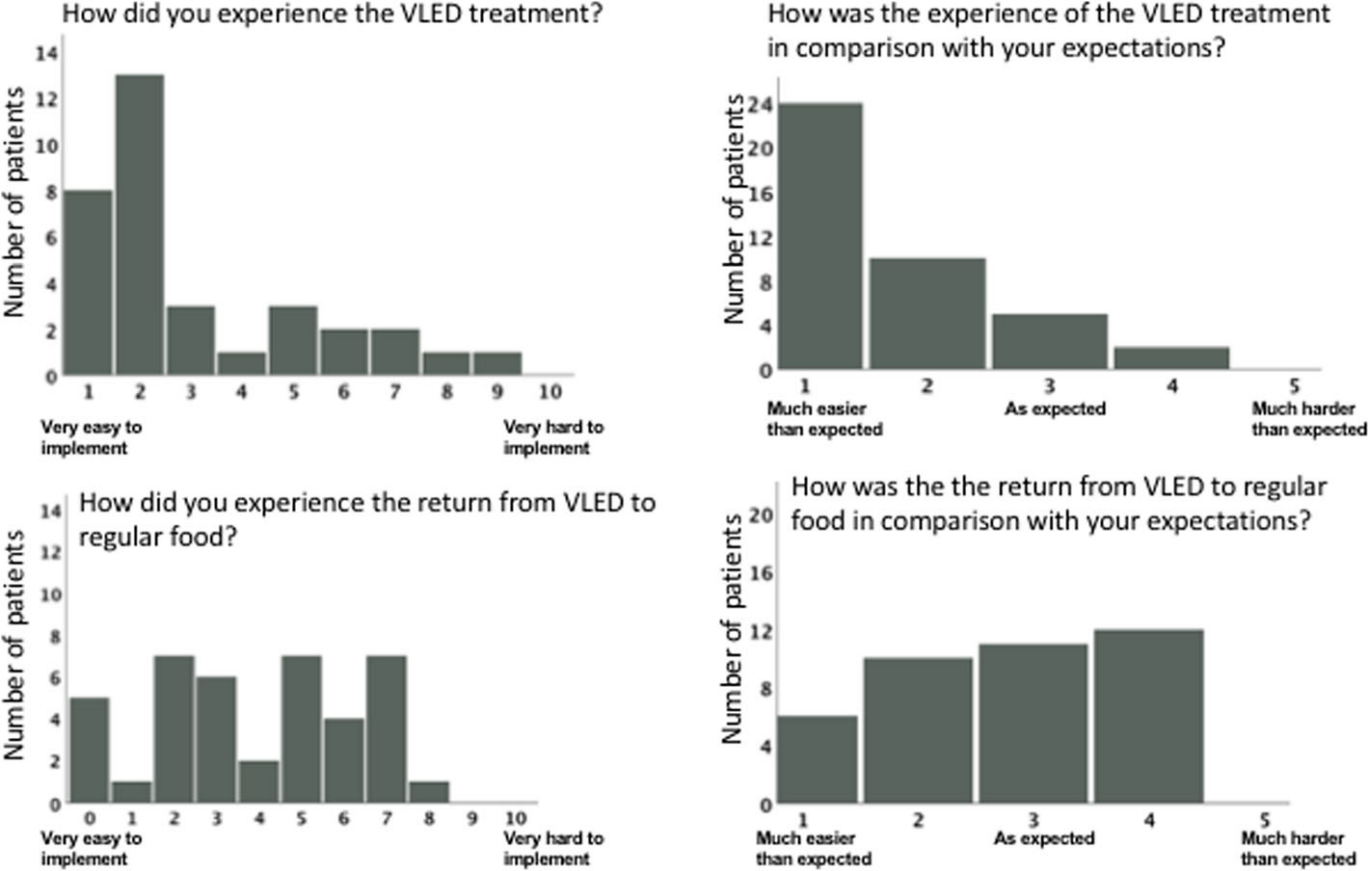
The patients’ experience of the very low energy diet (VLED) treatment and the transition from VLED to normal food

The treatment was generally well tolerated. Some patients experienced obstipation, freezing, loss of hair and hypotension during the strict VLED period and 12% a flare in psoriasis as previously mentioned. No serious adverse events occurred. When asked to score their experience of the VLED treatment between 1 (very easy to implement) and 10 (very hard to implement), the median score was 2 (IQR 1–3.5). When asked to score their experience of the VLED treatment in comparison to what they had expected, 83% (*n* = 34) answered “much easier than expected” or “easier than expected”. The patients generally scored their experience of the transition from VLED to normal food as harder. The median score was 4 (IQR 2–6) and totally 56% (*n* = 23) answered “as expected” or “harder than expected” (Fig. 3).

## Discussion

This study aimed to prospectively investigate the association between VLED weight loss treatment and disease activity in patients with PsA and obesity. We found significant improvement of the disease activity in joints, entheses and skin, reduction of CRP, PLT and parameters assessing function at the 6 months follow-up. The primary endpoint, patients with MDA, increased from 29.3% at baseline to 53.7% at 6 months (*p* = 0.002). PSARC was reached by 46% of the patients, and the ACR 20, 50 and 70 responses were 51%, 34% and 7% respectively. As a comparison, randomized controlled trials of TNF inhibitors on PsA have typically reported ACR 20 and 50 responses of 50–60% and 30–40% respectively, whereas the reported ACR 20 and 50 responses to placebo have been 15–25% and 0–5% [31, 32]. Reduction in weight correlated in a dose-response manner with higher ACR 20 response rate and greater reductions in DAS28CRP and HAQ. The treatment which resulted in a substantial weight loss of in median 18.6% of the baseline weight was well tolerated with only few and mild side effects and was perceived as feasible by a majority of the patients. Further, we found that a higher BMI at baseline was associated with increased disease activity and poorer self-reported function. The present study thus supports the hypothesis that obesity is a promotor of disease activity in PsA.

The mechanisms linking PsA occurrence and disease activity with obesity has not been fully clarified. Both immunological, biomechanical and behavioural mechanisms are likely to be involved in the interplay between PsA and obesity. PsA, obesity and atherosclerosis are conditions characterized by chronic inflammation, mediated via partly common immunologic pathways. Tumour necrosis factor α (TNFα), interleukin (IL)23, IL17, IL6 and IL1β are all key cytokines in the pathogenesis of PsA. The white adipose tissue (WAT) can produce the same cytokines (TNFα, IL-23, IL-17, IL-6, IL-1β) in addition to both pro-inflammatory adipokines (resistin, fetuin-A, chemerin, leptin) and adipokines viewed as primarily anti-inflammatory (adiponektin, omentin) [33]. In obese individuals, WAT is infiltrated by immune cells, such as M1-type macrophages, dendritic cells, IL17 producing T-lymphocytes (Th17) and B-lymphocytes [34]. Moreover, in obesity, there is a persistent over-production of the pro-inflammatory cytokines and adipokines, which may fuel the autoimmune inflammation in PsA [34, 35]. Interestingly, there is also evidence that localized inflammation in fat pads inside joints, juxta-posed to entheses and underneath psoriatic skin lesions may play a local pathophysiological role in PsA and psoriasis [36]. Elevated levels of leptin and resistin and lower levels of adiponectin in serum adjusted for BMI have been shown in patients with PsA and psoriasis in comparison with healthy controls. Elevated serum leptin and resistin have also been associated with an increased burden of skin and joint inflammation [37, 38].

Biomechanical stress may be another factor linking obesity with PsA. PsA is characterized by enthesitis, which is a chronic inflammation at attachment sites of tendons and ligaments, anatomical locations often subject to mechanical stress [39]. The weight load of obesity leads to both increased mechanical stress and risk of local microdamage. In the general population, obesity is also associated with pathology of entheses and tendons, in both upper and lower extremities [40]. In PsA, repeated occupational lifting of heavy loads and exposure to injury or bone fracture has been associated with onset of inflammatory arthritis in subjects with psoriasis [41, 42]. Psoriasis is also known to develop at sites of trauma, the so-called Köbner response [43]. Studies on animal models for spondyloarthritis (TNF^ΔARE^ and DBA/1 mice) have provided further proof for the importance of mechanical stress, by showing that unloading of the hind limb or tail suspension prohibited enthesitis and new bone formation in these mice [44].

Behavioural factors can also be a link between PsA and obesity. Pain and skin disease can induce a vicious cycle of lowered physical activity and over-eating, leading to obesity and further reduction of the mobility and physical activity [8, 45]. Efficient weight loss treatment could help reverse this process. Patients with PsA are at increased risk of developing CV and thromboembolic disease [12, 13]. In addition, obesity has been shown to be associated with poorer response and adherence to TNF inhibitor therapy [17, 46]. We recommend that weight loss treatment should be considered as complementary to pharmacological treatment in patients with PsA and obesity, since weight loss may improve both disease activity, effect of drug treatment and CV risk profile.

The reduced disease activity seen in the PsA patients after the VLED treatment in the present study could be due to a combination of mechanisms: metabolic effects associated with dietary energy restriction, reduction of pro-inflammatory mediators produced by the WAT, reduced loading of joints and entheses, especially in the back and lower limbs and reduced pain sensitivity. In situations of negative energy balance cells switch to utilize non-hepatic glucose, ketone bodies and free fatty acids as energy source [47]. This has a dampening and pro-apoptotic effect on activated T lymphocytes, which primarily depend on aerobic glycolysis [48]. Energy restriction and weight loss is also associated with increased levels of glucocorticoids and adiponectin and reduced levels of IL-6, TNFα and leptin in serum [49, 50]. In the current study, a negative energy balance was mainly present during the 12–16 weeks of strict VLED treatment. After this time point, only six patients (15%) had a further weight loss, whereas the rest of the patients had an increase in weight between the 3 and 6 months visits, indicative of a positive energy balance. The patients will be followed for 2 years to determine the relation between BMI and disease activity when the patients are no longer in a hypocaloric condition.

Obesity is associated with increased pain sensitivity in inflammatory rheumatic diseases, osteoarthritis and fibromyalgia [45]. In the current study, the change in composite scores for disease activity after weight loss was mainly driven by a reduction in the number of tender joints and entheses and by lowered patients’ VAS for pain and global disease activity, although there was also a reduction in parameters reflecting inflammation, such as CRP, PLT, swollen joints count and BSA.

There is one prior study which has prospectively studied the effects of weight loss in patients with PsA and obesity or overweight starting treatment with a TNF-inhibitor. After 6 months treatment, the patients with a successful weight loss (≥ 5%) had a higher rate of achieving MDA than those without a successful weight loss (MDA 50% vs 23%; *p* < 0.001) [17]. The patients had higher disease activity at baseline than in the present study, but the weight loss, which was achieved by a hypocaloric diet, was lower. One earlier randomized trial studied the effects of low-energy diet (LED) treatment (800–1000 kcal/day) compared with an ordinary healthy all-round diet in patients with psoriasis and obesity (30 vs. 30) and reported improvement of DLQI and cardiovascular risk profile in the LED arm, but only a trend towards improvement of Psoriasis Area and Severity Index (PASI) after 16 weeks. The weight loss was however correlated with the reduction of PASI. The beneficial effects were sustained at 64 weeks follow-up [51, 52]. Positive effects on psoriasis have also been reported retrospectively by patients after bariatric surgery in earlier studies [53, 54].

There are some limitations of the study. First, the present study lacks a control group. The success of the weight loss treatment is reliant on the patients’ determination to follow the given instructions and refrain from intake of energy-containing foods and beverages other than the VLED and non-caloric beverages during the initial strict period of 12–16 weeks. To be effective, VLED treatment must be based on the patients’ active decision and randomization between VLED and care-as-usual was therefore considered unsuitable. Second, no blinding of the assessors was made. Third, a high disease activity was not an inclusion criterion of the study, which may have affected the results. In fact, 29% of the patients had already MDA at baseline. Nevertheless, the study was able to show significant association between weight loss and reduced disease activity. Fourth, no documentation of the side effects (obstipation, freezing, loss of hair and hypotension) was done; hence, no exact data on the occurrence can be presented. No serious adverse event occurred however during the study. Fifth, the study was relatively short in duration, and it is indeed a great challenge to achieve long-term weight loss. The patients will however be followed during 24 months.

Strengths of the present study are the prospective design and the powerful intervention that resulted in a substantial weight loss due to excellent adherence to the dietary regime. The study demonstrates that adding a weight loss treatment to the conventional pharmacologic treatment in patients with PsA and obesity may lead to benefits including lowering of several measures of disease activity along with the weight reduction.

## Conclusions

Weight loss treatment with VLED in patients with PsA and obesity was associated with significant improvement of disease activity in joints, entheses and skin at 6 months follow-up. A larger weight loss resulted in more improvement in a dose-response manner. The treatment was effective, safe and well tolerated. In addition, we could demonstrate association between higher BMI and increased disease activity at baseline. The patients will be followed during 24 months to study long-term treatment effects. The study supports the hypothesis that obesity is involved in the pathophysiology of PsA.

## Additional file

Additional file 1: **Table S1.** Disease activity and function before and after weight loss treatment in the 29 patients with PsA who did not have minimal disease activity at baseline. (DOCX 16 kb)

## Abbreviations

ACR: American College of Rheumatology
BMI: Body mass index
BSA: Body surface area
CASPAR: Classification for Psoriatic Arthritis
CRP: C-reactive protein
cs/b DMARD: Conventional synthetic/biologic disease modifying anti-rheumatic drug
CV: Cardiovascular
DAPSA: Disease Activity in PSoriatic Arthritis
DAS28CRP: Disease Activity Score using 28 joint counts based on CRP
DLQI: Dermatology Life Quality Index
ESR: Erythrocyte sedimentation rate
HAQ: Health Assessment Questionnaire
Hb: Haemoglobin
IL: Interleukin
IQR: Interquartile range
MDA: Minimal disease activity
MetS: Metabolic syndrome
NSAID: Non-steroidal anti-inflammatory drug
PLT: Platelet count
PsA: Psoriatic arthritis
PsARC: Psoriatic Arthritis Response Criteria
*r*_S_: Spearman’s correlation
TNFi: Tumour necrosis factor inhibitor
TNFα: Tumour necrosis factor α
VAS: Visual analogue scale
VLED: Very low energy diet
WAT: White adipose tissue
WBC: White blood cell count
