# Supplementary material for: Weight loss improves disease activity in patients with psoriatic arthritis and obesity: an interventional study
Source: Arthritis Res Ther. 2019 Jan 11;21:17. doi: 10.1186/s13075-019-1810-5 (PMC6330463; doi:10.1186/s13075-019-1810-5)
Supplement: Supplementary file 1 — Table S1. Disease activity and function before and after weight loss treatment in the 29 patients with PsA who did not have minimal disease activity at baseline. (DOCX 16 kb) [file 13075_2019_1810_MOESM1_ESM.docx]

**Table S1** Disease activity and function before and after weight-loss treatment in the 29 patients with PsA who didn´t have minimal disease activity at baseline.

|  | **Baseline**  Median (IQR)  Min- Max | **6 months**  Median (IQR)  Min- Max | **p-value** |
| --- | --- | --- | --- |
| **Weight, kg** | 105.0 (95.8-115.8)  84-124 | 82.1 (75.9-90.9)  61.3-104.3 | <0.001 |
| **BMI, kg/m^2^** | 36.0 (34.5-39.3)  32.6-45.8 | 29.8 (27.2-31.7)  23.1-37.0 | <0.001 |
| **Waist circumference, cm** | 117 (108-122)  103-135 | 95 (90-101)  81-116 | <0.001 |
| **CRP, mg/L** | 5 (3- 11)  1-50 | 5 (2-9)  1-50 | 0.135 |
| **Hemoglobin, g/L** | 138 (130-146)  116-168 | 136 (131-144)  118-161 | 0.156 |
| **WBC, 10^9^/L** | 6.2 (5.4-7.6)  4.3-15.2 | 6.0 (4.9-7.6)  (3.8-12.7) | 0.112 |
| **PLT, 10^9^/L** | 271 (208-316)  174-444 | 264 (196-299)  156-402 | 0.001 |
| **Tender joints 68, score** | 8 (3-18)  0-30 | 6 (1-8)  0-19 | <0.001 |
| **Swollen joints 66, score** | 0 (0-2)  0-5 | 0 (0-0.5)  0-1 | 0.040 |
| **VAS Patients global disease activity, mm** | 45 (31-69)  18-93 | 23 (10-60)  0-95 | 0.002 |
| **VAS Pain, mm** | 57 (26-70)  16-95 | 28 (14-60)  0-95 | 0.003 |
| **VAS Fatigue, mm** | 62 (49-72)  12-94 | 30 (8-49)  0-98 | <0.001 |
| **DAS28CRP, score** | 3.4 (2.9-4.3)  2.2-5.6 | 2.9 (2.0-3.5)  1.3-4.5 | <0.001 |
| **DAPSA, score** | 20 (12.6-33.2)  6.7- 46 | 12.1 (6.4-22.4)  0.9- 35.2 | <0.001 |
| **Leeds enthesitis index** | 3 (0-4)  0-6 | 2 (0-3)  0-4 | 0.003 |
| **BSA, %** | 0.5 (0-2.5)  0-7 | 0.5 (0-2.0)  0-4 | 0.031 |
| **HAQ, score** | 0.75 (0.50- 1.19)  0-2.63 | 0.38 (0.19- 0.88)  0-1.88 | 0.001 |
| **DLQI, score** | 1 (0-5.5)  0-17 | 1 (0-4)  0-20 | 0.292 |

BMI = Body Mass Index, BSA = Body Surface Area, CRP = C- reactive protein, DAPSA = Disease Activity in PSoriatic Arthritis, DAS28CRP = Disease Activity Score using 28 joint counts based on CRP, DLQI = Dermatology Life Quality Index, PLT = platelet count, WBC = white blood cell count
